# Glutamate Chemical Exchange Saturation Transfer (GluCEST) Magnetic Resonance Imaging of Rat Brain With Acute Carbon Monoxide Poisoning

**DOI:** 10.3389/fneur.2022.865970

**Published:** 2022-05-19

**Authors:** Yuan Xu, Zerui Zhuang, Hongyi Zheng, Zhiwei Shen, Qilu Gao, Qihuan Lin, Rong Fan, Liangping Luo, Wenbin Zheng

**Affiliations:** ^1^Department of Radiology, The First Affiliated Hospital of Jinan University, Guangzhou, China; ^2^Department of Neurosurgery, The Second Affiliated Hospital of Shantou University Medical College, Shantou, China; ^3^Department of Radiology, The Second Affiliated Hospital of Shantou University Medical College, Shantou, China; ^4^Philips Healthcare China, Beijing, China

**Keywords:** carbon monoxide poisoning, magnetic resonance imaging-high field, glutamate, delayed neuropsychiatric syndrome, brain

## Abstract

**Objectives:**

To evaluate the diagnostic and prognostic values of glutamate chemical exchange saturation transfer (GluCEST) magnetic resonance imaging as a quantitative method for pathogenetic research and clinical application of carbon monoxide (CO) poisoning-induced encephalopathy combined with the proton magnetic resonance spectroscopy (^1^H-MRS) and the related histopathological and behavioral changes.

**Methods:**

A total of 63 Sprague–Dawley rats were randomly divided into four groups. Group A (*n* = 12) was used for animal modeling verification; Group B (*n* = 15) was used for magnetic resonance molecular imaging, Group C (*n* = 15) was used for animal behavior experiments, and Group D (*n* = 21) was used for histopathological examination. All the above quantitative results were analyzed by statistics.

**Results:**

The peak value of carboxyhemoglobin saturation in the blood after modeling was 7.3-fold higher than before and lasted at least 2.5 h. The GluCEST values of the parietal lobe, hippocampus, and thalamus were significantly higher than the base values in CO poisoning rats (*p* < 0.05) and the ^1^H-MRS showed significant differences in the parietal lobe and hippocampus. In the Morris water maze tests, the average latency and distance were significantly prolonged in poisoned rats (*p* < 0.05), and the cumulative time was shorter and negatively correlated with GluCEST.

**Conclusion:**

The GluCEST imaging non-invasively reflects the changes of glutamate in the brain *in vivo* with higher sensitivity and spatial resolution than ^1^H-MRS. Our study implies that GluCEST imaging may be used as a new imaging method for providing a pathogenetic and prognostic assessment of CO-associated encephalopathy.

## Introduction

Carbon monoxide (CO) poisoning is a common gas-poisoning disease in clinical practice, which can lead to the highest mortality among all types of acute gas poisonings (1, 2). In CO poisoning, a large amount of CO enters the bloodstream through the lungs and binds to the hemoglobin in the blood to form carboxyhemoglobin (HbCO). This reduces oxygen diffusion and transportation, leading to acute hypoxia (3).

The central nervous system has a large demand for oxygen (4). The symptoms of acute CO poisoning are proportional to the degree of hypoxia and are manifested as dizziness, headache, attention deficit, muscle weakness, nausea, vomiting, and even coma (3, 5). In clinical practice, most patients with acute CO poisoning often recover very soon; however, about 10–45% of patients may have a series of neurological symptoms after 20–40 days of pseudo recovery, which is known as CO poisoning-related delayed encephalopathy (6–8). The main clinical manifestations include dementia, mental and consciousness disorders, pyramidal, extrapyramidal, and local cerebral cortical dysfunction (3, 9). The occurrence has no direct connection with acute conditions (6, 10). The pathogenesis of CO poisoning-related delayed encephalopathy has not yet been elucidated, and numerous hypotheses have been proposed for various pathogenesis, such as cytotoxic damage mechanism (11, 12), immune dysfunction (13), and neurotransmitter disorder mechanism (14). Due to its high disability rate and heavy nursing burden, early diagnosis of CO poisoning-related delayed encephalopathy is of great significance. Numerous studies have shown that the content of glutamate in the brain increases significantly after acute CO poisoning (14–17). Glutamate acts on postsynaptic receptors to overload the intracellular calcium levels, resulting in damage to cells and apoptosis (16). Craniocerebral injuries mainly occur in the form of apoptosis in patients with CO poisoning-related delayed encephalopathy (14, 18).

Magnetic resonance molecular imaging is a promising, quantitative, and *in vivo* method for noninvasively dynamic monitoring of small molecular changes in brains. The proton magnetic resonance spectroscopy (^1^H-MRS) is currently the most common way to detect glutamate, together with a variety of other neurochemicals in the brain (19, 20). But it has the ability neither to detect the low concentration of glutamate in the brain, nor to differentiate glutamate from glutamine and gamma-aminobutyric acid due to resonance overlap (21, 22).

Glutamate chemical exchange saturation transfer (GluCEST) magnetic resonance imaging (MRI) is a recently developed MR molecular imaging technology for the label-free measurement of glutamate *in vivo* by its exchangeable amino protons using the chemical saturation transfer (CEST) technology (23). CEST offered clinical molecular imaging and molecular diagnosis when combined with magnetic resonance technology to image both anatomical structure and molecular-level function. CEST can also help evaluate pathology and therapeutic efficacy (24). The chemical exchange effects of glutamate via the amine group showed a CEST peak at 3.0 ppm, and the concentration of glutamate in the brain was sufficiently high for glutamate detection by CEST (25). GluCEST MRI has good spatial and temporal resolutions and has been applied to the central nervous system of healthy volunteers (25–28). The distribution of glutamate from GluCEST between the gray and white matter is similar to the results of positron emission tomography imaging using radionuclide-targeted glutamate receptor reagents, which is also positively related to the glutamate concentration as measured by ^1^H-MRS. GluCEST could be used to determine and confirm the epileptic foci of patients with non-lesion temporal lobe epilepsy, and to observe the histological and neurochemical changes of the brain tissues after traumatic encephalopathy and diffuse axonal injury, which is helpful for clinical diagnosis and treatment (29–31). In this study, we explored the potential utility of GluCEST MRI for monitoring the changes of glutamate in the brain with CO poisoning, so that doctors could evaluate the brain injury after CO poisoning dynamically and non-invasively, hoping to realize the early diagnosis of CO poisoning-related delayed encephalopathy.

## Materials and Methods

### Animal Models

All animal study protocols were approved by the Animal Ethics Committee of the University following the Animal Research: Reporting of *In Vivo* Experiments (ARRIVE) guidelines as well as in accordance with the U.K. Animals (Scientific Procedures) Act of 1986 and its associated guidelines.

Sprague Dawley rats (*n* = 63; male, 200–300 g) were purchased from Shantou University Animal Center and divided into four groups. Group A (*n* = 12) was used for animal modeling verification, Group B (*n* = 15) was used for magnetic resonance molecular imaging, Group C (*n* = 15) was used for animal behavior experiment, and Group D (*n* = 21) was used for histopathological examination.

All of the rats were anesthetized with 4% isoflurane mixed with oxygen to minimize the pain until righting reflex of the rats disappeared before and during operations. The CO-poisoned treatments were given high-purity CO gas (120 ml/kg body weight) by intraperitoneal injection (32–34), while the control treatments were injected with the same dose of air. Since the aim of our study is to investigate the changes in glutamate metabolism in the living brain after carbon monoxide poisoning, the dead rats due to poison were replaced by additional rats and all death data would be excluded.

### Verification of Animal Models

**Figure 1:**
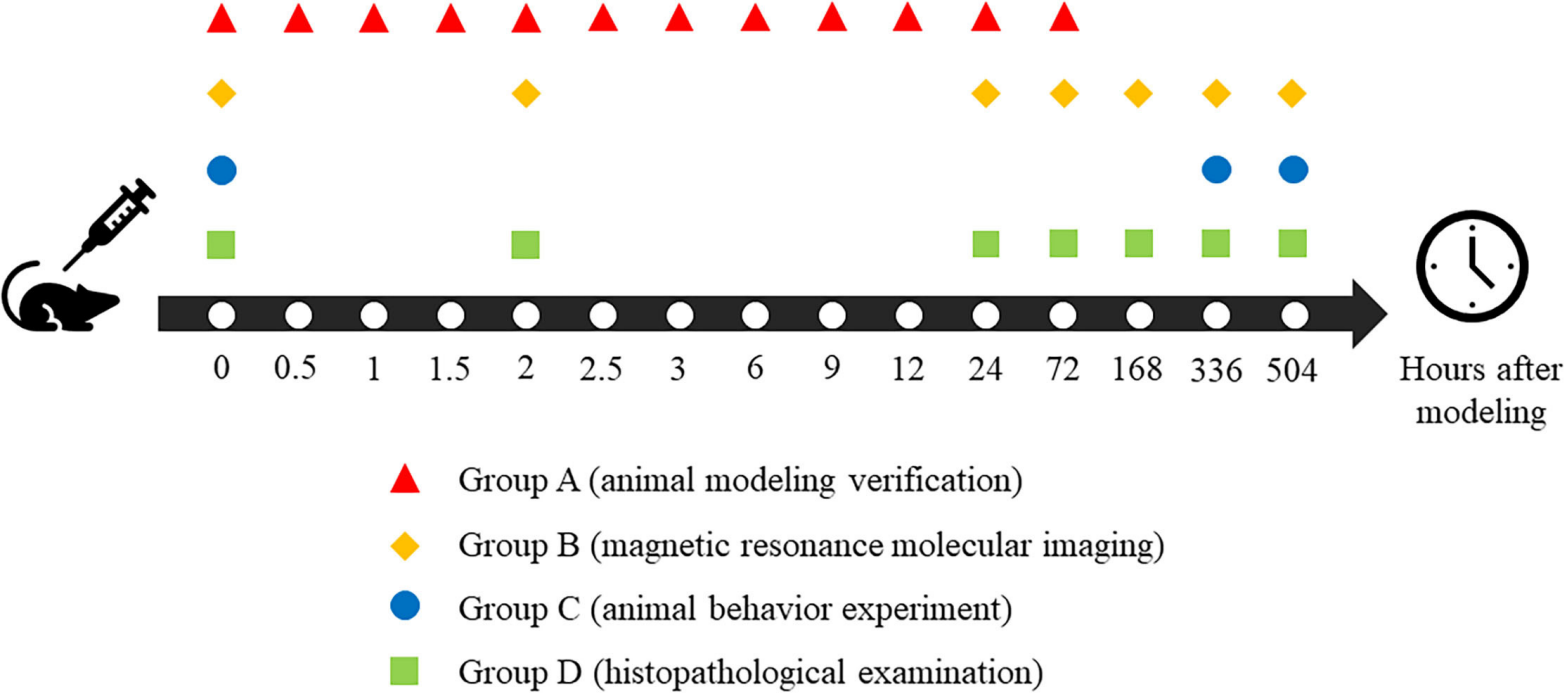
Time points arrangement of all rats in different groups.

To ensure the reliability and stability of the animal models, we first confirmed the formation of brain injury in the animal receiving CO treatment before conducting other experiments (Group A). Blood samples were taken from the left ventricle according to the 12 timepoints, as shown in Figure 1. Anesthesia with 2.0–2.5% isoflurane and 1 L/min oxygen were maintained throughout the entire process in all rats after induction anesthesia with 4% isoflurane and 1 L/min oxygen to minimize pain in rats. The carboxyhemoglobin saturation (HbCO%) is a quantitative parameter of verification. It was measured in left ventricle blood using spectrophotometry (35).

### MR Data Acquisition

All of the magnetic resonance studies were acquired using a 7.0 T animal magnetic resonance scanner (Agilent VnmrJ 3 Imaging, Palo Alto, CA) using a transmitter/receiver body coil (diameter = 160 mm). All of the rats in Group B participated in magnetic resonance scanning according to the schedule in Figure 1 and were anesthetized with 2.0–2.5% isoflurane and 1 L/min oxygen mixture throughout the acquisition process. The respiratory frequency of rats was monitored using a small animal respiratory monitor (MR-compatible small animal monitoring & gating system, Model 1030, SA Instruments, Stony Brook, NY).

The three-dimensional (axial, sagittal, and coronal) localization images were scanned at first to determine the location of the rat brain and to help adjust the position with the following parameters: repetition time/echo time = 24 s/2 ms, the field of view (FOV) = 50 mm × 50 mm, 929 × 2 matrix, 1.0 mm thickness without a gap. Then we obtained the three-dimensional T2-weighted images (T_2_WI) using a fast spin echo sequence with the following parameters: repetition time = 3,000 ms, FOV = 35 mm × 35 mm, 2562 × 56 matrix, 2.0 mm thickness, and 0.2 mm gap.

Before GluCEST MRI acquisition, we chose an axial slice with the largest hippocampal area as the target and corrected the static magnetic field (B_0_) with three-dimensional gradient filling. Then we modified the high-order gradient filling current based on the derived B_0_ maps and further adjusted the radio frequency field (B_1_) and center frequency in the pre-scanning. The inhomogeneity of B_0_ and B_1_ were modified according to the B_0_ and B_1_ maps from the same target slice. For B_0_ correction, the water saturation shift referencing technique (36) was used with the parameters as following: TE/TR = 64/2,000 msec, RARE factor = 8, frequency resolution increments =0.1-ppm and frequency coverage = ±1 ppm. GluCEST sequence was obtained by using frequency-selective continuous wave pulse for pre-saturation and echo plane imaging for image acquisition. The CEST saturation frequencies ranged from−5 to +5 ppm with an interval of 0.2 ppm and the parameters were as follows: B_1_5 _=_ .9 μT (250Hz), saturation time = 2s, FOV = 35 mm × 35 mm, TR = 4,100 ms, TE = 42 ms, 1281 × 28 matrix size, 2 average, 2.0-mm thickness, and the total acquisition time is 9 mins.

Following the GluCEST acquisition, ^1^H-MRS was acquired using the point-resolved spectroscopy sequence acquisition. The regions of interest (ROIs) were chosen based on three-dimensional T_2_WI images. The ^1^H-MRS sequence parameters were as follows: TR = 5,000 ms, data matrix = 3203 × 20, ROIs were located at the bilateral hippocampus, parietal cortex, and thalamus; the voxel size is 2.0 mm × 3.0 mm × 10.0 mm.

The quality of MR data is closely related to the homogeneity of the magnetic field, so repeated shimming and B_0_ mapping were carried out during the scanning process to ensure the quality of data with a linewidth of <20 Hz.

### Animal Behavior Observation and Experiment

Throughout the process of modeling, the symptoms and behavioral changes of rats 2 h after modeling were observed and recorded. Moreover, the Morris water maze (MWM) test including the positioning navigation experiment and space exploration experiment were performed before and after modeling (14 and 21 days) of Group C to preliminarily evaluate the effects of CO poisoning on the brain.

All of the rats in Group C were moved into the animal behavioral laboratory in advance to adapt to the environment and reduce tension and discomfort. In the positioning navigation experiment, rats were allowed to swim for up to 120 s to a hidden platform separately 4 times a day for 5 consecutive days and to rest on the platform for 60 s each time regardless of whether they found the platform or not. The space exploration experiment was carried out on the5^th^ day following the positioning navigation experiment.

### Data Post-Processing

The original data collected by the GluCEST sequence were analyzed by a program written using the MATLAB R2013b Software (MathWorks, Natick, MA). The formula for calculating glutamate concentration showed as follows:


$$\begin{array}{l} MTRsaym(3.0ppm)=MTR(+3.0ppm)-MTR(-3.0ppm)\\ \;=\frac{Ssat(-3.0ppm)-Ssat(+3.0ppm)}{So} \end{array}$$

*(MTR*_*asym*_ *, asymmetric magnetization transfer rate; MTR, magnetization transfer rate; S*_*sat*_ *, signal strength after applying saturated pulse; S*_0_ *, signal strength after not applying saturated pulse)*

Then the contrast of GluCEST was corrected according to the inhomogeneity of B_0_ and B_1_. In this study, we used GluCEST% to describe MTRsaym (3.0ppm). The GluCEST% (in direct proportion to glutamate concentration) was mapped to the anatomical image as pseudo-color to get the image, and calculated as the relative glutamate concentration in the ROIs.

The original data of ^1^H-MRS were performed in the LCModel and LCMgui software (v. 6.2–4E; S.W. Provender). The appropriate parameters of 1H-MRS were selected for automatic linear fitting and data processing analysis. The relative concentrations of metabolites were obtained by using the concentration of water in rat brain tissue as an internal reference. In the estimated using the LCModel fitting, only metabolite concentration values that have a standard deviation (SD) <20 were considered trustable and used in the analysis.

The video of the MWM experiment was analyzed by EthoVision® XT (Noldus, Wageningen, Netherlands). The latency time, the latency distance, and the cumulative time in the correct quadrant could be obtained in the positioning navigation experiment and the space exploration experiment.

### Histopathological Examination of Animal Brain Slices

The rats in Group D were anesthetized until their righting reflex disappeared. After cardiac perfusion, with 4% polyformaldehyde solution, their brain tissue was removed, according to the timepoints described in Figure 1, for 48 h and then immobilized in paraffin solution. Fixed wax blocks were cut on a microtome (RM2125, Leica Biosystems, German) until the layers (hippocampus, parietal cortex, and thalamus) appeared. The brain was then continuously sliced at every 5 microns. All of the sections were dyed with hematoxylin and eosin. Finally, all of the sections were dehydrated, rendered transparent, and fixed with resin. Images were collected with the help of a microscope after all pathological sections were dried (IM50, Leica, Olympus Corporation, Japan).

### Statistical Methods

All of the results were analyzed by IBM SPSS Statistics 16.0 software. The results for each quantitative parameter were expressed as the mean ± SD. One-way analysis of variance and Student's *t*-test was applied to compare among and between groups when the data were normally distributed, and the variance was homogeneous. Otherwise, a non-parametric test was used instead. Pearson correlation analysis was applied when data were normally distributed, and Spearman correlation analysis was applied when data were not normally distributed. The level of *p* < 0.05 was considered to be statistically significant.

## Results

### Detection of Blood HbCO%

**Figure 2:**
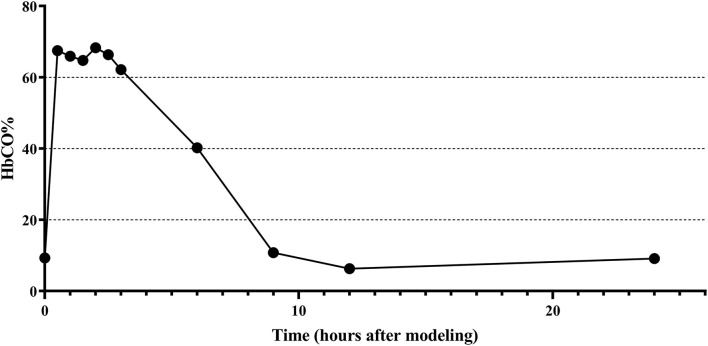
Changes of HbCO% in left ventricular blood of rats before and after CO exposure.

The changes of HbCO% in the left ventricular blood of rats before and after 0.5, 1, 1.5, 2, 2.5, 3, 6, 9, 12, 24, and 72 h of CO exposure are shown in Figure 2, where the HbCO% in the blood increased rapidly, plateaued, and then gradually decreased from the plateau stage to near the pre-exposure level after intraperitoneal injection of high purity CO. The peak value of HbCO% in the blood after exposure was 7.3-fold higher than before exposure. The highly elevated HbCO% level lasted for approximately 2.5 h after exposure.

### Magnetic Resonance Examinations of Rat Brain

**Figure 3:**
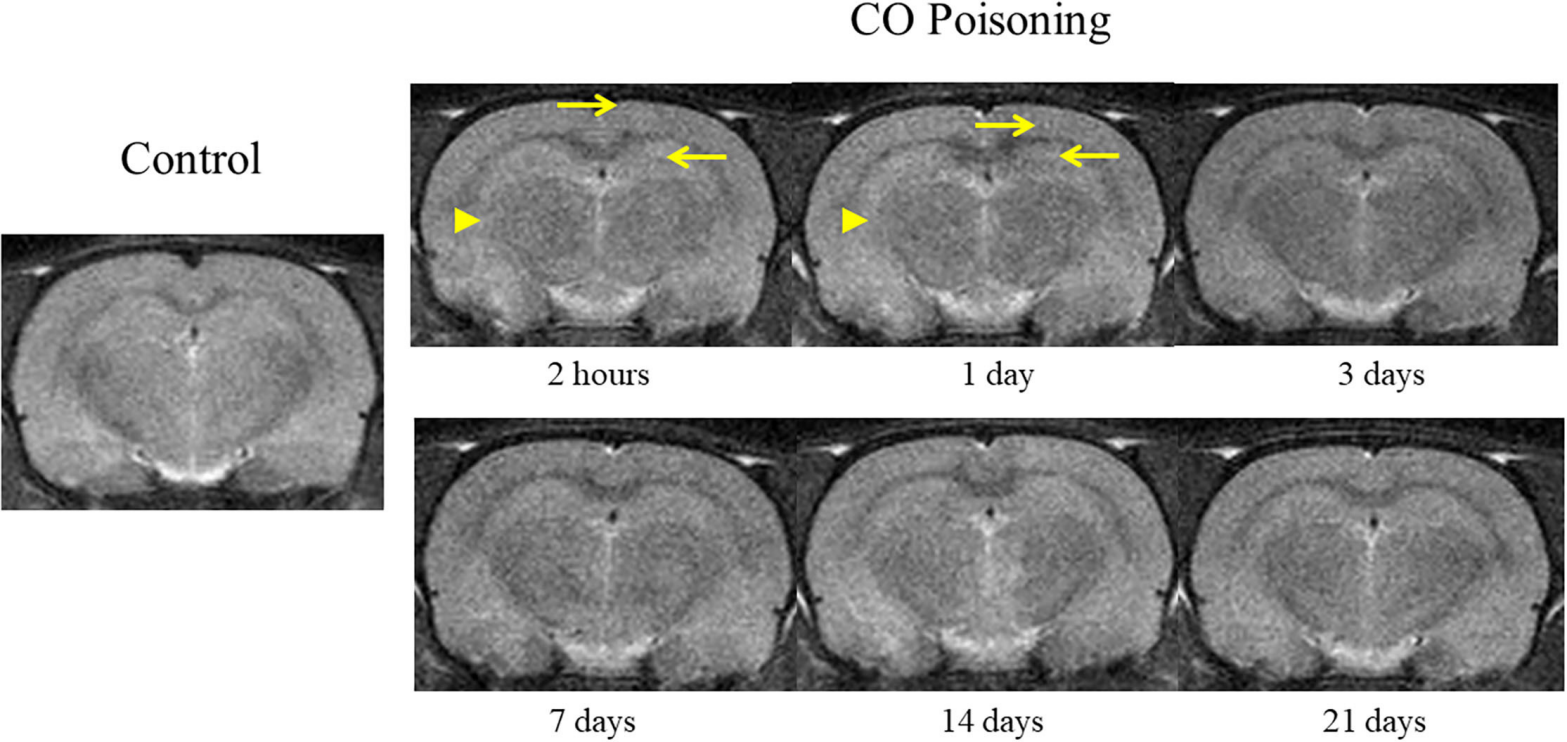
Representative T_2_WI images at different time points before and after CO exposure.

The changes in brain T_2_WI were not obvious after CO poisoning. Punctate hyperintensity (pointed by arrow; Figure 3) could be seen in the cortex and hippocampus. After poisoning, the boundary between the gray and the white matter was blurred (pointed by arrowhead; Figure 3) in the first 24 h. However, these changes were difficult to identify and could not be determined qualitatively and quantitatively.

The magnetic resonance examinations data were normally distributed and their variance was homogeneous, so Student's *t*-test was applied to compare the poisoned group and the control group at different time points.

**Figure 4:**
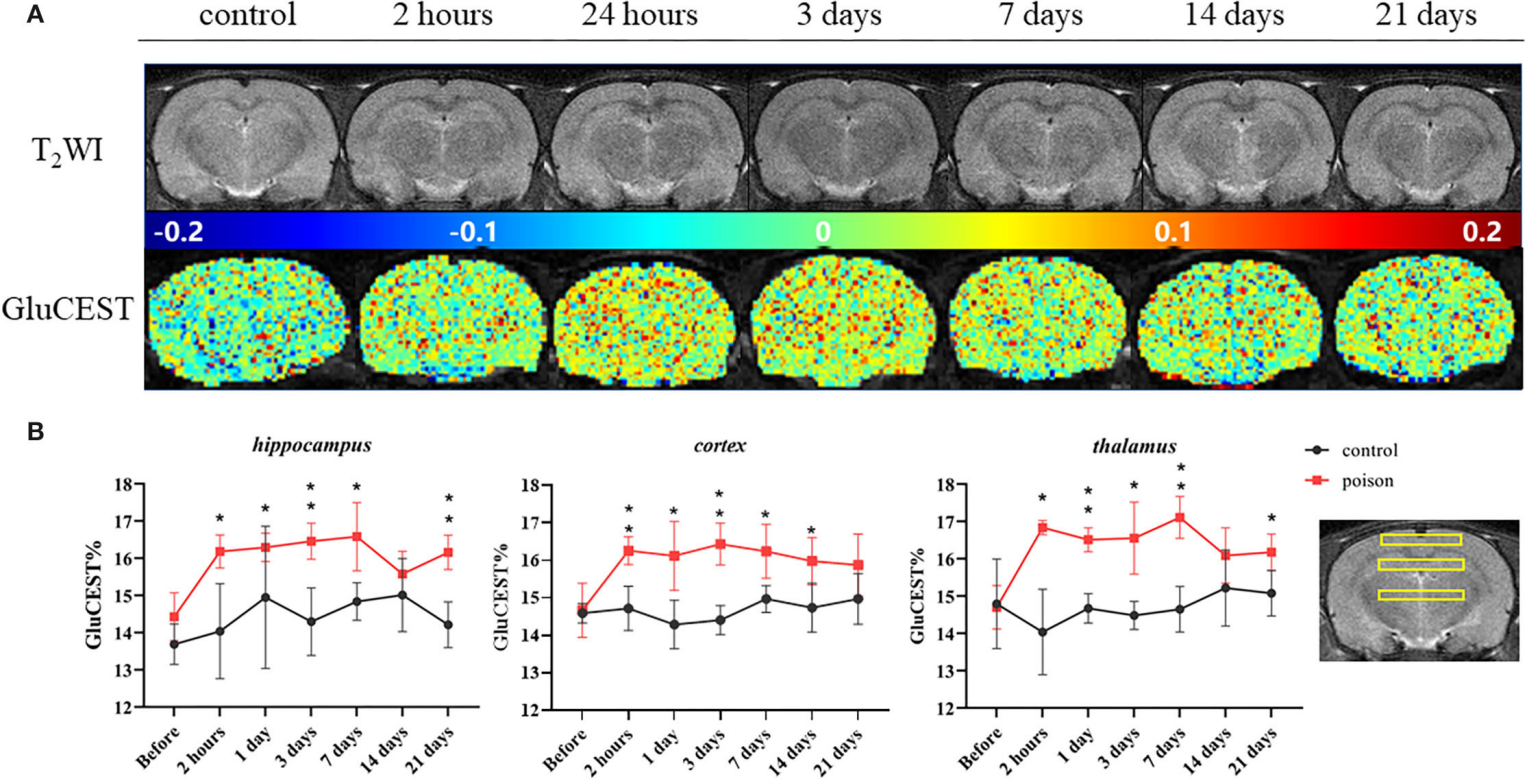
Representative T_2_WI images and GluCEST map of the target-level **(A)** and regional GluCEST values **(B)** at different time points before and after CO exposure. *p < 0.05, **p < 0.01.

Before modeling, there was no significant difference in the GluCEST% value between Group B1 and B2. After modeling, the GluCEST% value of the poisoning group (B2) was significantly higher than the control group (B1); the difference was statistically significant (Figure 4). The differences in the three ROIs (bilateral hippocampus areas, cortical areas, and thalamus areas) continued to the 7^th^ day after CO-poisoning. In the hippocampus area, the value of GluCEST% increased continuously to the peak value on the 7^th^ day after CO-poisoning; the value in the cortical areas and thalamus areas reached their first peak in 24 h.

**Figure 5:**
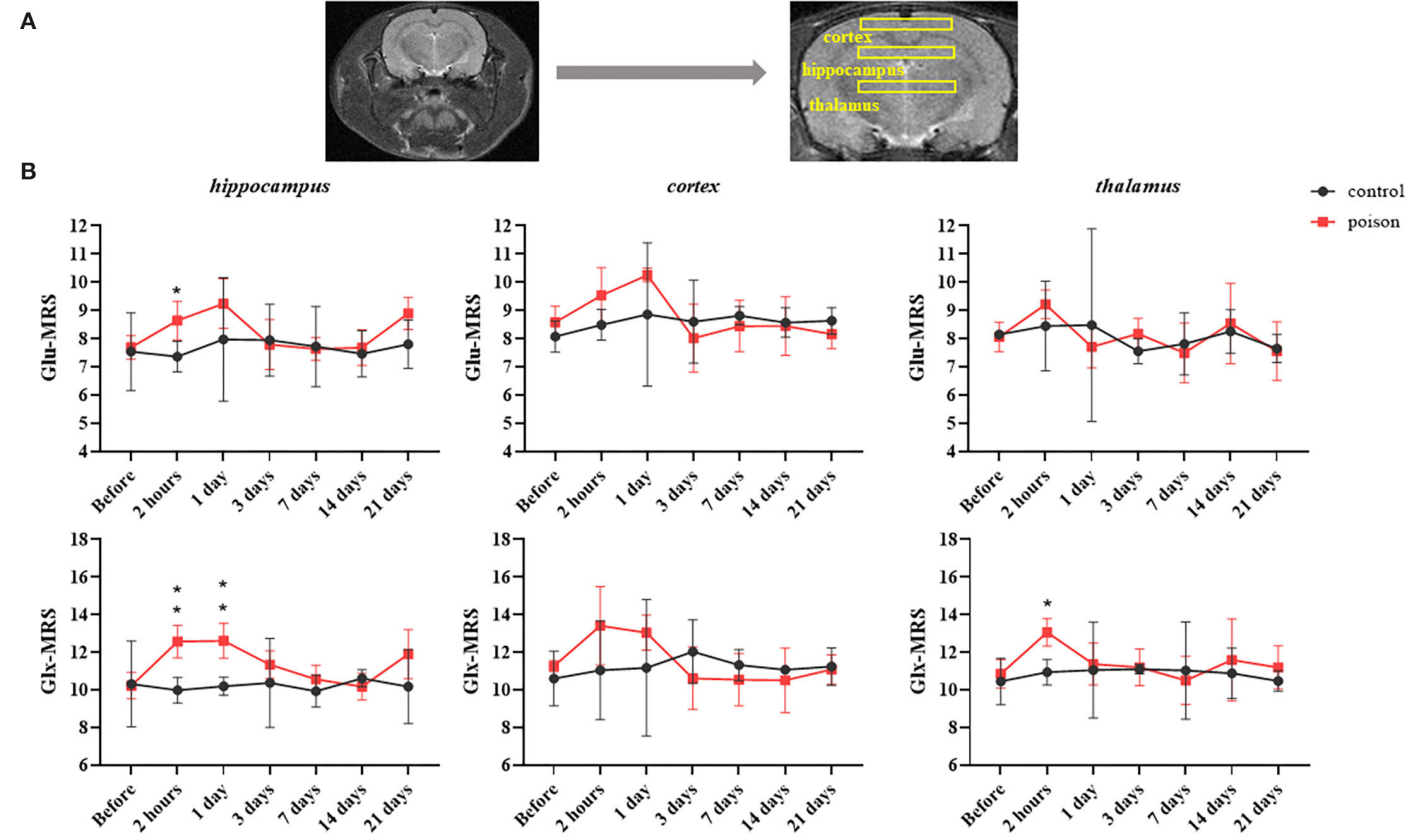
Regions of interest in the T_2_WI for ^1^H-MRS **(A)** and regional data **(B)** from ^1^H-MRS at different time points before and after CO exposure. *p < 0.05, **p < 0.01.

Compared with that before the poisoning, the value in Figure 5 of Glu and Glu+Gln (Glx) from ^1^H-MRS increased statistically in bilateral hippocampus and thalamus areas after CO exposure, and the differences in the hippocampus and thalamus persisted 24 and 2 h after modeling respectively. But the Glu and Glu+Gln increased in bilateral cortical areas after CO exposure without statistical differences.

### Animal Behavioral Analysis

The poisoned rats showed a redder mucosa around their auricle and mouth than the control 5 mins after intraperitoneal injection of high purity CO. They also had weaker hind limbs. Some rats showed shortness of breath, convulsions, coma, and even death. There were no significant abnormal changes in the control rats after injection.

In the animal behavioral data, the escape latency time data were normally distributed and their variance was homogeneous, so we applied Student's *t*-test to compare the poisoned group and the control group. Meanwhile, the rest of all the other animal behavioral data were not normally distributed, so the nonparametric test was used.

**Figure 6:**
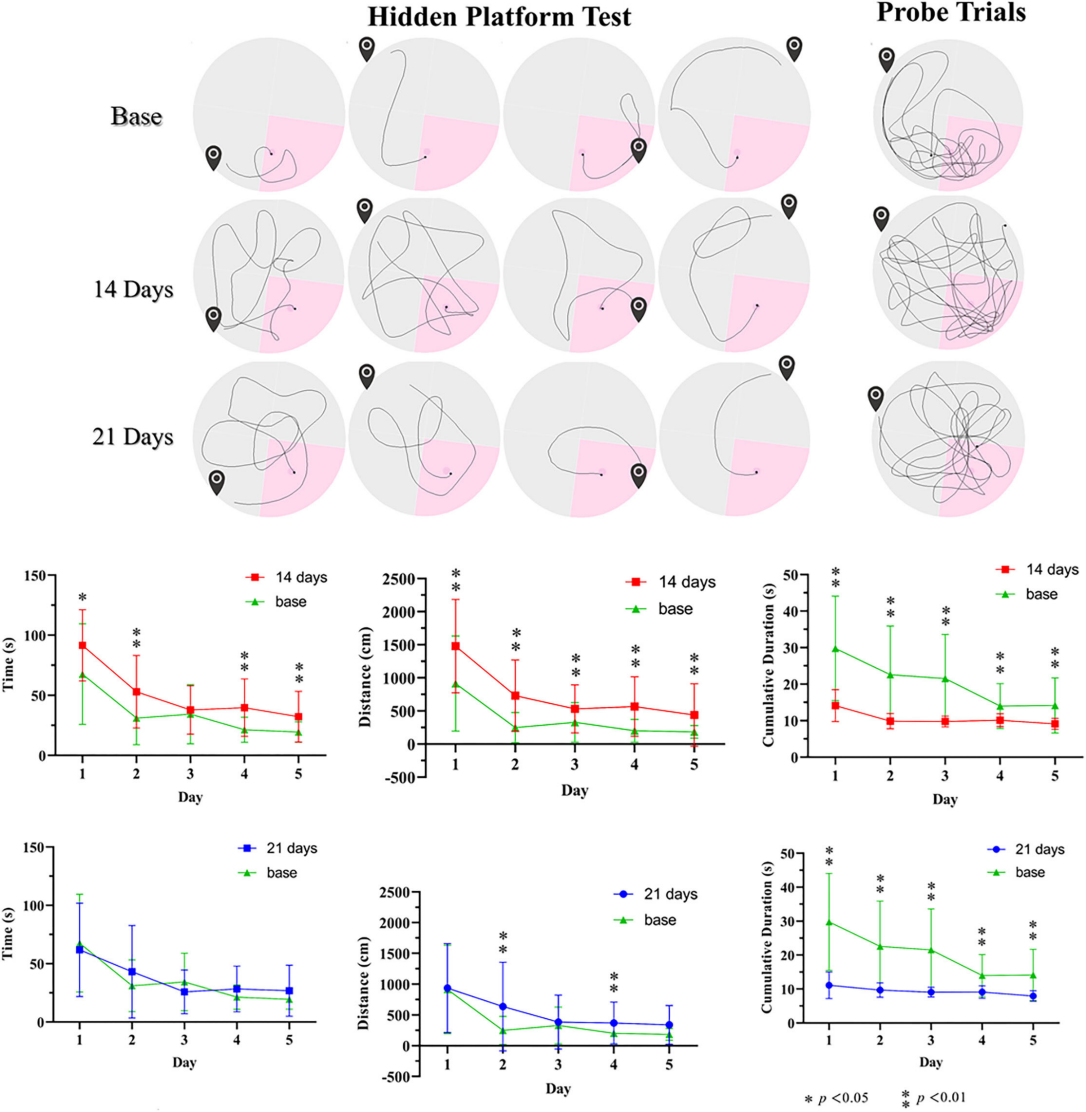
Illustration of pathways before and after CO poison in the Morris water maze and related data. * *p* < 0.05, ** *p* < 0.01.

We studied behavioral changes of the CO-exposed effectiveness on Group C rats via MWM (Figure 6). In the orientation navigation experiment, the poisoned rats of 14 days after modeling had significantly longer escape latency time vs. baseline (the control treatments). The poisoned rats had longer swimming distances. Furthermore, the cumulative time in the correct quadrant of the poisoned rats in both poisoning time points was less than baseline. In the space exploration experiment, the cumulative swimming time in the target quadrant of the poisoned rats in both poisoning time points was less than that at baseline; there were no statistical differences in swimming velocity.

### Correlations Between the GluCEST and MWM Related Results

Since the magnetic resonance examinations data and the escape latency time data were normally distributed and their variance was homogeneous, Pearson correlation analysis was applied. Meanwhile, the rest of all the other animal behavioral data were not normally distributed, so we used Spearman correlation to analyze the other correlations.

**Figure 7:**
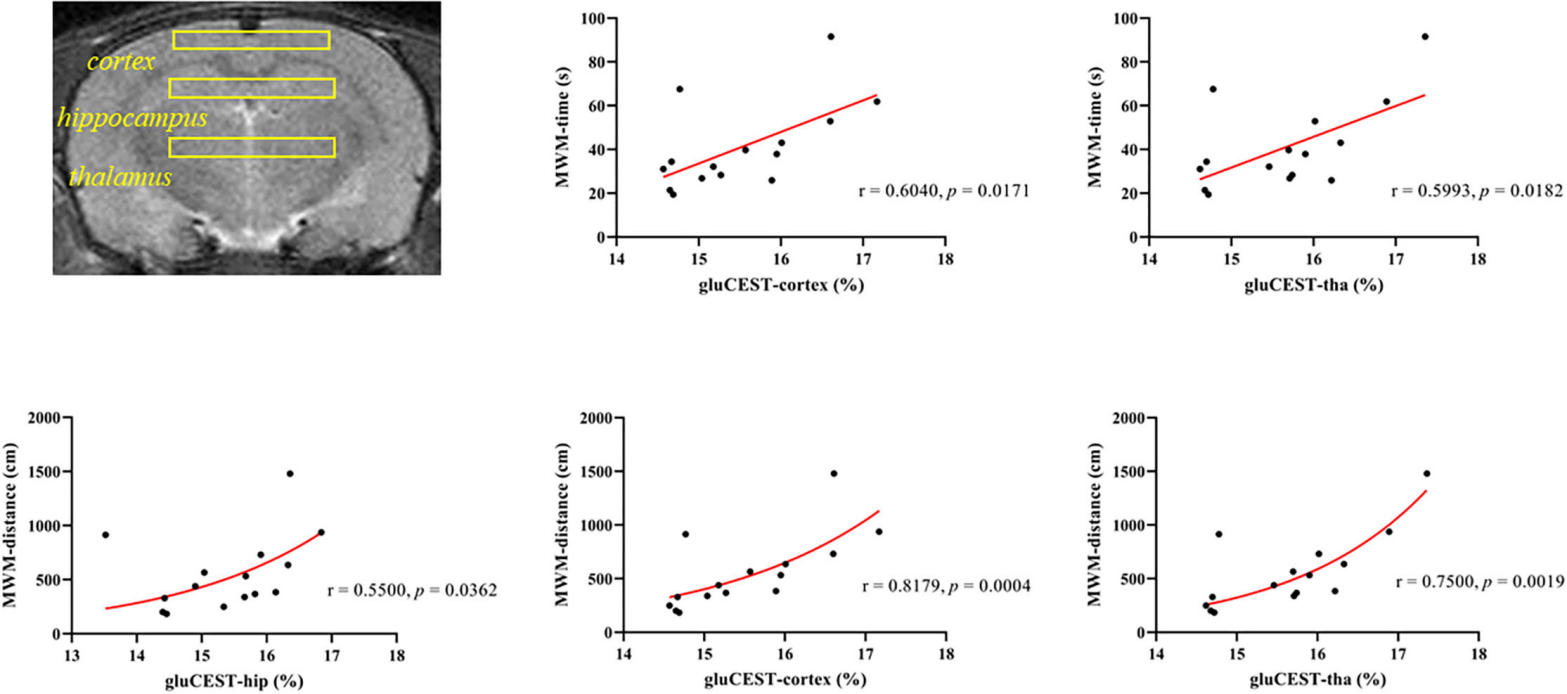
Correlations between the cumulative time from the Morris water maze and the GluCEST% value after CO poisoning.

Since 21 days after poisoning was chosen as the end point of our study, we conducted a correlation analysis between the GluCEST data of the three ROIs, respectively, on the 21st day after poisoning and the average MWM data of the 21 days group. Figure 7 showed correlations between GluCEST% values and MWM-related results. There was a positive correlation between the latency time and GluCEST% of parietal cortex (ρ = 0.618, *p* = 0.014) and thalamus (ρ = 0.518, *p* = 0.048) when the strong positive correlations between swimming distances and GluCEST% of parietal cortex (ρ = 0.818, *p* < 0.001) and thalamus (ρ = 0.751, *p* = 0.001) were observed.

### Histopathological Findings

**Figure 8:**
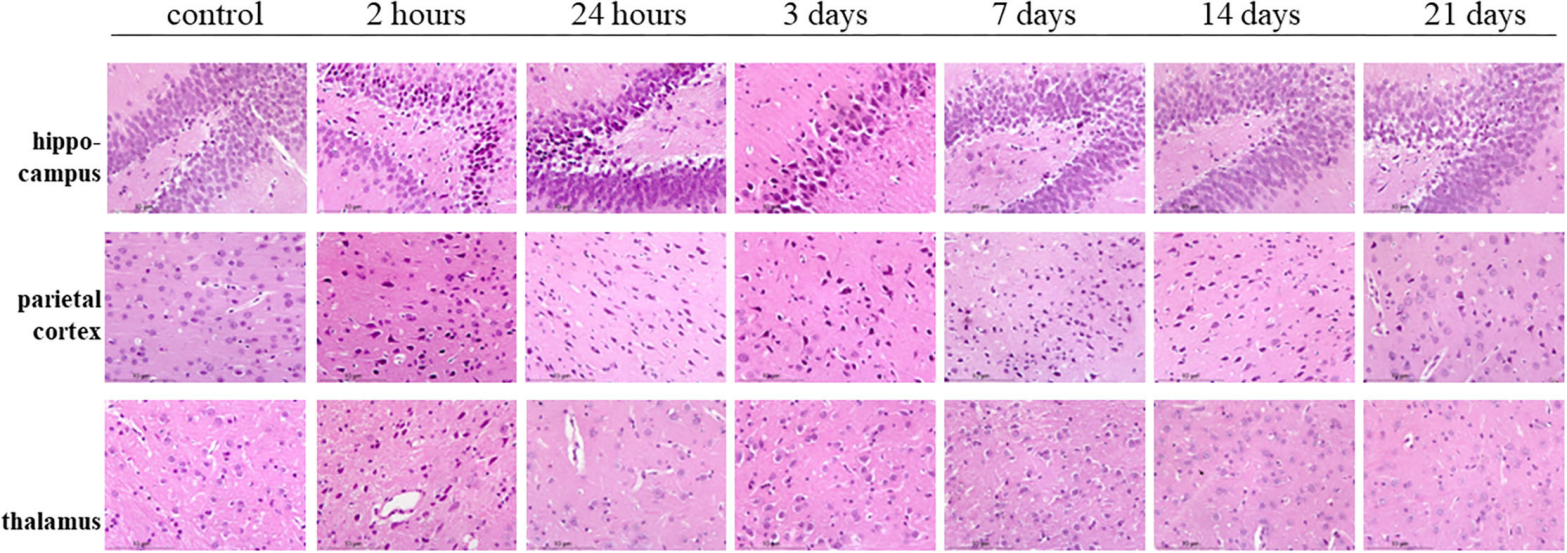
Hematoxylin–eosin staining of the hippocampus, parietal cortex, and thalamus in rat brains at multiple time points after exposure.

Hematoxylin–eosin staining showed the bilateral hippocampus areas and parietal lobe areas of the poisoned rats showed pyknosis, hyperchromatic cytoplasm, and disappearance of central Nissl bodies within 3 days after exposure. The lesions mentioned above decreased significantly 7, 14, and 21 days after exposure. The thalamic areas of the poisoned rats were only obvious 2 h after exposure, and the lesions were then significantly reduced over time (Figure 8).

## Discussions

Our study for the first time, investigated the ability of GluCEST MRI to monitor the spatial and temporal changes in glutamate concentration in rats with CO poisoning-related delayed encephalopathy. The GluCEST% contents were significantly increased in the bilateral hippocampus, parietal cortex, and thalamus after CO exposure via CEST evaluation. Meanwhile, the CEST MRI findings were consistent with the ^1^H-MRS measurement, which showed that the Glu and Glu+Gln values were higher in the bilateral hippocampus and the parietal cortex of the poisoned rats than in pre-injection of CO.

Piantadosi et al. performed micro-dialysis of the cerebral cortex and hippocampus in CO-poisoned rats and observed increased Glu and hydroxyl radical contents in the cerebral cortex and hippocampus in CO-poisoned rats (14). There could be CO-related cardiovascular effects and other indirect effects mediated by CO. Raub and Benignus described the interaction between CO and the nervous system, suggesting that the increased glutamate content was related to delayed amnesia (non-acute) (37). Our results supported the findings of the previous studies, and we investigated the changes in glutamate in the brain up to 21 days after CO poisoning.

Glutamate is the main excitatory amino acid in the brain, and there are several causes for increased glutamate production. First, the low-energy state caused by hypoxia induces the reverse transport of glutamate transporter and stimulates the release of glutamate in the synaptic glutamatergic neurons (38). Second, lysed or dead cells release cytosolic glutamate (39). Third, the reverse cystine/glutamate antiporter function of neuronal-glial cells surrounds the neurons (40). Fourth, the presence of impaired regulation of glutamate receptors (40). These mechanisms lead to a positive feedback loop of cell death and accumulation of glutamate neurotransmitters promoting the development of excitatory neurotoxicity. The excitatory neurotoxic effects of glutamate were mainly shown as (1) intracellular Ca^2+^ overload leading to mitochondrial damage and biofilm damage inducing energy failure and cell death (14), (2) increased free radical formation and lipid peroxidation (41), and (3) abnormal activation of the nitric oxide synthase–nitric oxide (NOS–NO) system (41). This damage eventually leads to necrosis or apoptosis and induced changes in glutamate receptors that might overlap the pathogenesis of nervous system diseases such as epilepsy and Parkinson's disease thereby leading to CO-related delayed neuropsychiatric sequelae (42, 43).

Our study used the MWM to evaluate learning and memory in the rat model. The MWM is a spatial learning test for rodents (44) that has been used in the study of traumatic brain injury and aging for assessing cognitive deficits (45, 46). The average latency and distance traveled of the rats after CO exposure was longer than the baseline before poisoning suggesting that CO poisoning significantly reduced the short-term spatial learning and memory of rats. The cumulative time in the correct quadrant of the poisoned rats was shorter than the baseline further verifying the rat performance. Negative correlations between the GluCEST% and the cumulative time in the target quadrants further suggested that glutamate might correlate with the CO-related delayed encephalopathy.

This study does have a few limitations. The longest time point of the GluCEST measurement was 21 days. Given the onset period of CO poisoning-related delayed encephalopathy could be longer, a longer observation window will help draw a more comprehensive understanding of glutamate-based pathogenesis of CO poisoning-related delayed encephalopathy. Second, the GluCEST maps were very noisy. The sources of noise might be as follows: the inhomogeneity of the magnetic field, the complex compositions and structures of animal bodies, the breathing movement of animals, and so on. The following steps should be taken to improve the signal-to-noise ratio of GluCEST imaging in the following research: adjusting the homogeneity of a magnetic field by shimming, reducing the bandwidth, increasing the number of excitations, slice thickness, and field of view, and decreasing the matrix size. GluCEST is a new neuroimaging method to quantitatively assess CNS damage, and could non-invasively monitor changes in brain glutamate levels *in vivo*, which is promising for pathogenetic and prognostic assessment of CO-based encephalopathy. Our study may provide new insight into acute CO poisoning and help improve the early diagnosis of CO poisoning-related delayed encephalopathy.

## Data Availability Statement

The original contributions presented in the study are included in the article/supplementary material, further inquiries can be directed to the corresponding author/s.

## Ethics Statement

The animal study was reviewed and approved by the Animal Ethics Committee of Shantou University.

## Author Contributions

YX: conceptualization, methodology, software, investigation, formal analysis, writing—original draft, visualization, and writing—reviewing and editing. ZZ: conceptualization, methodology, and software. HZ: methodology and formal analysis. ZS: methodology, software, and formal analysis. QG, QL, and RF: investigation. WZ: conceptualization, methodology, writing—reviewing and editing, supervision, project administration, and funding acquisition. LL: supervision and writing—reviewing and editing. All authors contributed to the article and approved the submitted version.

## Funding

This study has received funding by the National Natural Science Foundation of China (Grant No. 81571627), Grant for Key Disciplinary Project of Clinical Medicine under the Guangdong High-level University Development Program, Joint Research Fund for Enterprise and basic and applied basic research Programs of Guangdong Province of China (Grant No. 2021A1515 220112), and the Natural Science Foundation of Guangdong Province of China (Grant No. 2021A1515 011179).

## Conflict of Interest

ZS was employed by Philips Healthcare China. The remaining authors declare that the research was conducted in the absence of any commercial or financial relationships that could be construed as a potential conflict of interest.

## Publisher's Note

All claims expressed in this article are solely those of the authors and do not necessarily represent those of their affiliated organizations, or those of the publisher, the editors and the reviewers. Any product that may be evaluated in this article, or claim that may be made by its manufacturer, is not guaranteed or endorsed by the publisher.
